# The association of white matter connectivity with prevalence, incidence and course of depressive symptoms: The Maastricht Study

**DOI:** 10.1017/S0033291722002768

**Published:** 2023-09

**Authors:** Anouk F. J. Geraets, Sebastian Köhler, Laura WM Vergoossen, Walter H. Backes, Coen D.A. Stehouwer, Frans RJ Verhey, Jacobus FA Jansen, Thomas T. van Sloten, Miranda T. Schram

**Affiliations:** 1 Department of Psychiatry and Neuropsychology, Faculty of Health, Medicine & Life Sciences, Maastricht University, Maastricht, the Netherlands; 2 Alzheimer Centrum Limburg, Maastricht University Medical Center+ (MUMC+), Maastricht, the Netherlands; 3 Department of Internal Medicine, Maastricht University Medical Center+ (MUMC+), Maastricht, the Netherlands; 4 Department of Radiology and Nuclear Medicine, Maastricht University Medical Center+ (MUMC+), Maastricht, the Netherlands; 5 Heart and Vascular Center, Maastricht University Medical Center+ (MUMC+), Maastricht, the Netherlands; 6 School for Mental Health and Neuroscience, Faculty of Health, Medicine & Life Sciences, Maastricht University, Maastricht, the Netherlands; 7 School for Cardiovascular Diseases (CARIM), Faculty of Health, Medicine & Life Sciences, Maastricht University, Maastricht, the Netherlands

**Keywords:** Brain connectivity, depression, depressive symptoms, epidemiology, magnetic resonance imaging, population-based cohort

## Abstract

**Background:**

Altered white matter brain connectivity has been linked to depression. The aim of this study was to investigate the association of markers of white matter connectivity with prevalence, incidence and course of depressive symptoms.

**Methods:**

Markers of white matter connectivity (node degree, clustering coefficient, local efficiency, characteristic path length, and global efficiency) were assessed at baseline by 3 T MRI in the population-based Maastricht Study (*n* = 4866; mean ± standard deviation age 59.6 ± 8.5 years, 49.0% women; 17 406 person-years of follow-up). Depressive symptoms (9-item Patient Health Questionnaire; PHQ-9) were assessed at baseline and annually over seven years of follow-up. Major depressive disorder (MDD) was assessed with the Mini-International Neuropsychiatric Interview at baseline only. We used negative binominal, logistic and Cox regression analyses, and adjusted for demographic, cardiovascular, and lifestyle risk factors.

**Results:**

A lower global average node degree at baseline was associated with the prevalence and persistence of clinically relevant depressive symptoms [PHQ-9 ⩾ 10; OR (95% confidence interval) per standard deviation = 1.21 (1.05–1.39) and OR = 1.21 (1.02–1.44), respectively], after full adjustment. On the contrary, no associations were found of global average node degree with the MDD at baseline [OR 1.12 (0.94–1.32) nor incidence or remission of clinically relevant depressive symptoms [HR = 1.05 (0.95–1.17) and OR 1.08 (0.83–1.41), respectively]. Other connectivity measures of white matter organization were not associated with depression.

**Conclusions:**

Our findings suggest that fewer white matter connections may contribute to prevalent depressive symptoms and its persistence but not to incident depression. Future studies are needed to replicate our findings.

## Introduction

Structural brain changes, such as brain atrophy (Barsky & Silbersweig, 2017; Disabato & Sheline, 2012; Drevets, Price, & Furey, 2008; Price & Drevets, 2010) and cerebral small vessel disease (Geraets et al., 2021; van Agtmaal, Houben, Pouwer, Stehouwer, & Schram, 2017), have been associated with the risk of depression in large population-based studies. However, both atrophy and cerebral small vessel disease represent overt and generally irreversible end-organ damage to the brain. Novel markers may compose new biomarkers at an earlier disease stage and may contribute to better understand the neuropathogenesis of depression. Previous studies have indicated that the structural organization of the white matter, also named white matter connectivity, may represent such a novel marker (Bullmore & Sporns, 2009; Rubinov & Sporns, 2010; Stam, 2010).

**Fig. 1.**
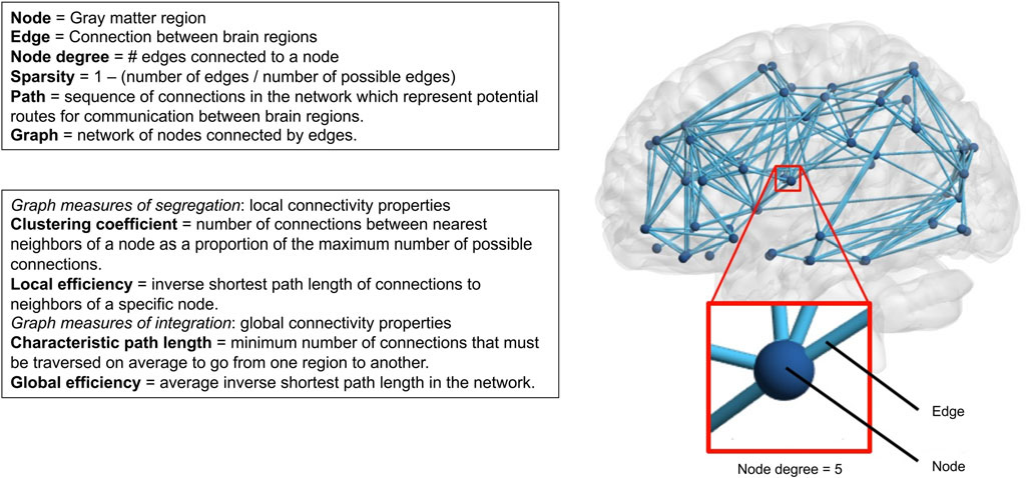
Overview of white matter connectivity measures.

White matter connectivity is responsible for efficient information exchange between brain regions. Alterations in one region may affect the function of other regions to which they are connected via white matter tracts. Diffusion-weighted magnetic resonance imaging (dMRI) allows quantification of the white matter connectivity using graph theory. In graph theory, the brain is represented as a graph, which is a network of nodes (gray matter regions) connected by edges (white matter connections). A simple representation of such a white matter network is shown in Fig. 1; with in the red square an enlarged node connected with edges to five other nodes.

Despite the increasing knowledge of brain alterations in depression (Gray, Müller, Eickhoff, & Fox, 2020; Koshiyama et al., 2020; Zhang et al., 2016), data on the association between white matter connectivity and depression on a population-based level are scarce. Clinical studies that examined whole-brain white matter networks have yielded mixed results (Gong & He, 2015). Two studies have reported disrupted global properties in patients with remitted depression in older individuals (Bai et al., 2012; Li et al., 2017). In addition, another clinical study has shown an association between some, but not all markers of white matter connectivity in individuals with a major depressive disorder (MDD) compared to healthy controls (Yao et al., 2019). In contrast, two other studies did not find an association between a MDD and global white matter connectivity measures (Korgaonkar, Fornito, Williams, & Grieve, 2014; Qin et al., 2014). These inconsistent results may be a result of differences in the determination of white matter connectivity and characteristics of the study population, including differences in age and depressive episode. All studies included small (*n* < 100), clinical samples, which hampers the generalization to the general population. Furthermore, only one small study that included older individuals with a remitted depression (*n* = 10) had a longitudinal design (Li et al., 2017).

Therefore, the aim of this study was to investigate whether different markers of white matter connectivity are associated with prevalence, incidence, and course of depression in a large population-based cohort. We hypothesized that markers of global white matter connectivity are altered in the prevalent, incident, and persistent depression, as expressed by fewer white matter connections and weaker organization of white matter networks.

## Method

### Study population and design

We used data from The Maastricht Study, an observational prospective population-based cohort study. The rationale and methodology have been described previously (Schram et al., 2014). In brief, the study focuses on the etiology, pathophysiology, complications and comorbidities of type 2 diabetes mellitus (T2DM), heart disease, and other chronic conditions, and is characterized by an extensive phenotyping approach. Eligible for participation were all individuals aged between 40 and 75 years living in the southern part of the Netherlands. Participants were recruited through mass media campaigns and from the municipal registries and the regional Diabetes Patient Registry via mailings. Recruitment was stratified according to known T2DM status, with an oversampling of individuals with T2DM, for reasons of efficiency. Baseline data were collected between November 2010 and December 2017. Magnetic resonance imaging (MRI) measurements were implemented from December 2013 onwards. Follow-up data was collected annually over a period of seven years, we used data currently available among 88.0% (year 1), 79.3% (year 2), 73.1% (year 3), 62.9% (year 4), 53.0% (year 5), 29.8% (year 6), and 11.9% (year 7) of the participants. The lower availability of data after the fifth year of follow-up is a result of the ongoing annual follow-up from year 6 onwards. The study has been approved by the institutional Medical Ethical Committee (NL31329.068.10) and the Minister of Health, Welfare and Sports of the Netherlands (Permit 131088-105234-PG). All participants gave written informed consent.

**Fig. 2.**
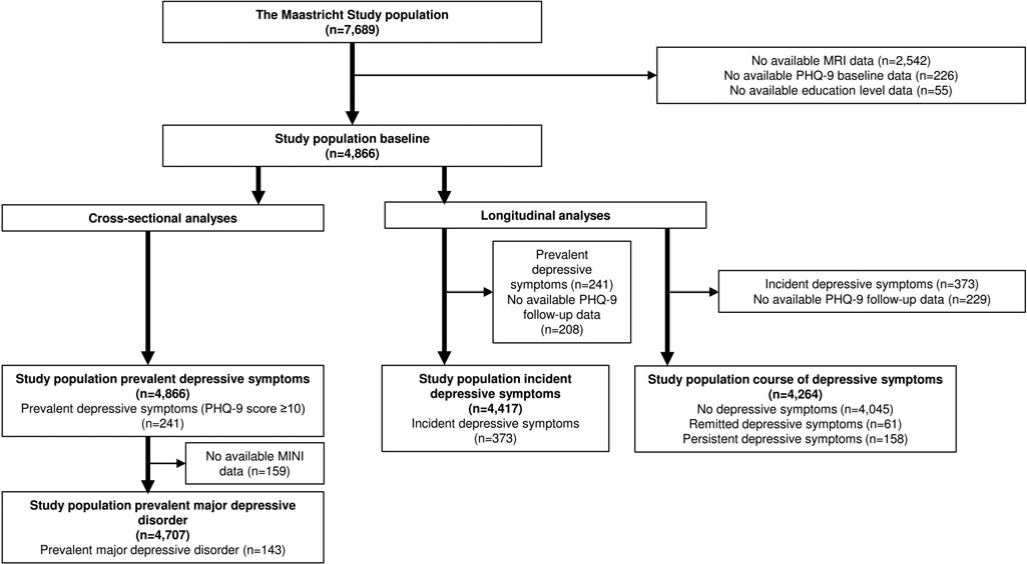
Flowchart of study population.

Figure 2 shows the flowchart of the study population. From the initial 7689 participants, baseline MRI data were available from *n* = 5147 participants. Of these, data on baseline Patient Health Questionnaire-9 (PHQ-9) and educational level were available in *n* = 4866. These 4866 participants were included in the cross-sectional analyses on prevalent depressive symptoms. Baseline data on baseline Mini-International Neuropsychiatric Interview (MINI) were available in *n* = 4707.

Next, two different study populations were composed for the longitudinal analyses. (1) For the analyses on incident depressive symptoms, we excluded participants with clinically relevant depressive symptoms at baseline (PHQ-9 ⩾ 10, *n* = 241) and those without PHQ-9 follow-up data (*n* = 208), resulting in a study population of 4417 participants with an average follow-up duration of 3.9 ± 1.0 years. 2) For the analyses on course of depressive symptoms, we excluded participants without PHQ-9 follow-up data (*n* = 229) and those with incident depressive symptoms (*n* = 373). This resulted in a study population of 4264 participants. The distribution of persistent and remitted cases during follow-up is shown in online Supplementary Fig. S2.

### Brain magnetic resonance imaging

Brain MRI was performed on a 3 Tesla MRI scanner (MAGNETOM Prisma-fit Syngo MR D13D; Siemens Healthcare, Erlangen, Germany) by use of a 64-element head coil for parallel imaging, as previously described (Vergoossen et al., 2020). More information about image acquisition and pre-processing is provided in the Supplemental Methods.

### Global node degree

Network analysis was performed using the Brain Connectivity Toolbox (version 2017-15-01) (Rubinov & Sporns, 2010) in MATLAB (Release, 2016), as described previously (Vergoossen et al., 2020). The node degree is the number of white matter connections (edges) to a certain gray matter brain region (node), see Fig. 1. The node degree is calculated for each atlas region, and the mean value is defined as the global node degree, which is a measure for the average number of edges connected to a region (node). In a network with a high global node degree, nodes are connected to many other nodes in the network (i.e. strong innervation).

In white matter connectivity analysis, connectivity matrices are based on tractography. As a result, not all pairs of nodes will have connections, which leads to ‘sparse’ connectivity matrices. In this study, a standard network was proportionally thresholded to a sparsity of 0.80, a commonly used value for proportional thresholding (Buchanan et al., 2020). Only the connections that were present in 20% of the edges with the highest occurrence over the individual networks will be included in the individual connectivity matrices, resulting in a weighted, undirected network with a sparsity close to the sparsity of the standard network. More information can be found in the online Supplemental Methods.

### Graph measures

The organization of the white matter was characterized using graph measures, e.g., clustering coefficient, local efficiency, characteristic path length, and global efficiency (Fig. 1). These graph measures describe the segregation and integration of the white matter networks. Measures of segregation describe the local connectivity properties of a network and comprise clustering and local efficiency. The clustering coefficient quantifies the number of connections between the nearest neighbors of a region as a proportion of the maximum number of possible connections (Watts & Strogatz, 1998). The local efficiency of a region is the inverse of the average shortest path connecting all neighbors of that region (Latora & Marchiori, 2001). We calculated the mean clustering coefficient and local efficiency of all regions per participant. Paths are sequences of connections in the network, which represent potential routes for communication between brain regions. Measures of integration describe the ease with which brain regions communicate in terms of paths and include characteristic path length and global efficiency. The characteristic path length is the minimum number of connections that must be traversed on average to go from one region to another (Watts & Strogatz, 1998). The global efficiency is the inverse of the average shortest path length calculated over the entire brain; thus, a high global efficiency reflects long paths between regions (Latora & Marchiori, 2001).

### Depression

Depressive symptoms were assessed by a validated Dutch version of the PHQ-9 (Kroenke, Spitzer, & Williams, 2001) both at baseline and during annual follow-up over seven years. More information about the PHQ-9 is provided in the Supplemental Methods. A cut-off score of ⩾10 is most often used as a dichotomous scoring system for defining clinically relevant depressive symptoms (Pettersson, Bostrom, Gustavsson, & Ekselius, 2015). Because there was a time lag between the baseline data collection and the MRI scan, the PHQ-9 score obtained closest to the MRI scan was chosen as the baseline score for each individual.

Prevalent depressive symptoms were defined as clinically relevant depressive symptoms at baseline (PHQ-9 ⩾ 10). Incident depressive symptoms were defined as no depressive symptoms at baseline (PHQ-9 < 10) and the presence of clinically relevant depressive symptoms on at least one follow-up moment (PHQ-9 ⩾ 10). Course of depressive symptoms was defined as (1) persistent depressive symptoms, i.e. clinically relevant depressive symptoms at baseline and on at least one follow-up moment (PHQ-9 ⩾ 10); (2) remitted depressive symptoms, i.e. clinically relevant depressive symptoms at baseline (PHQ-9 ⩾ 10) and no clinically relevant depressive symptoms during follow-up (PHQ-9 < 10); and (3) no depressive symptoms, i.e. no clinically relevant depressive symptoms at baseline and any of the follow-up examinations (no incident depressive symptoms; PHQ-9 < 10).

In addition, at baseline only, current and lifetime diagnosis of MDD was assessed by the MINI (Sheehan et al., 1998). The MINI is a short diagnostic structured interview, used to assess the presence of MDD in the preceding two weeks, according to the Diagnostic and Statistical Manual of Mental Disorders, Fourth Edition. We also used the MINI to assess the age of MDD onset. The MINI was conducted by trained staff members at the research center.

### General characteristics and covariates

General characteristics and covariates were measured at baseline. Educational level (low, intermediate, high), history of cardiovascular diseases (CVD), smoking status (never, current, former), alcohol consumption (none, low, high), physical activity (Harada, Chiu, King, & Stewart, 2001), and healthy diet score (van Lee et al., 2012) were assessed by questionnaires (Schram et al., 2014), as described previously. Cerebrovascular event (CVE) was assessed by questionnaire (Rose, 1962) and MRI. History of CVE was defined as present if confirmed by questionnaire or MRI. We measured T2DM, height, weight, waist circumference, office blood pressure, plasma and lipid profile as described elsewhere (Schram et al., 2014). Medication use was assessed in a medication interview where the generic name, dose, and frequency were registered. Because of an MRI scanner update that took place 16 February 2015, analyses are adjusted for MRI date before or after this update.

### Statistical analyses

All statistical analyses were performed by use of the Statistical Package for Social Sciences version 25.0 (IBM Corp., 2017). General characteristics of the study population were evaluated using independent *t* tests, Mann–Whitney *U* tests or χ^2^ tests. We inversed the values of node degree, clustering coefficient, local efficiency, and global efficiency [i.e. multiplying them by − 1 to reflect structural dysconnectivity (i.e. higher scores = more dysconnectivity), which eases the interpretation of risk associations. In cross-sectional analysis, we investigated the associations of global average node degree and graph measures with depressive symptoms (PHQ-9 score), presence of clinically relevant depressive symptoms (PHQ-9 ⩾ 10) and current MDD (MINI) by use of negative binominal regression and logistic regression analyses, respectively.

We used Cox proportional hazard regression analyses to assess the associations of global average node degree, regional average node degree, and graph measures with incident depressive symptoms (PHQ-9 ⩾ 10) in those without depressive symptoms at baseline, with time-in-study as time axis. Multinomial logistic regression analyses were used to investigate whether these markers were associated with a persistent or remitted course of depressive symptoms, with ‘no depressive symptoms’ as a reference group. Associations were adjusted for potential confounders in three models: model 1, age, sex, educational level, MRI update, T2DM and average node degree (for graph measures); model 2, additionally adjusted for waist circumference, total-to-high-density lipoprotein cholesterol ratio, lipid-modifying medication, systolic blood pressure, antihypertensive medication, history of CVD, history of CVE; model 3, additionally adjusted for smoking status and alcohol use.

Several additional analyses for prevalent and course of depressive symptoms were performed. To reduce potential misclassification of participants with subthreshold depression, we (1) additionally adjusted for use of antidepressant medication at baseline, (2) excluded participants who used antidepressant medication at baseline from the control group, and (3) excluded participants who had an MDD diagnosis or missing MDD data at baseline from the control group. We additional adjusted for a healthy diet and physical activity separately because these data were not complete, i.e., missing in respectively 230 and 394 participants. To investigate whether the associations could be explained by vascular brain damage, we additionally adjusted for white matter hyperintensity volume and intracranial volume. Although the PHQ-9 assessment the closest to the MRI date was taken as baseline, there could still be a time lag of a maximum one year between PHQ-9 and MRI assessment. To investigate whether this time lag influenced our results, we additionally adjusted for it. Because previous studies found regional differences in white matter connectivity (Korgaonkar et al., 2014; Qin et al., 2014), we performed post-hoc additional analyses on regional node degree measures for specific regions that have been related to depression-related structural alterations (limbic system, anterior cingulate cortex, medial temporal lobe and prefrontal cortex). A two-sided P-value<0.05 was considered statistically significant.

## Results

### General characteristics of the study population

At baseline, 241 participants (5.0%) had prevalent depressive symptoms (PHQ-9 ⩾ 10), and 143 (2.9%) had MDD. During 17 406 person-years of follow-up, 373 participants (8.4%, incidence rate = 21,4 per 1000 person-years) developed incident depressive symptoms (PHQ-9 ⩾ 10). Table 1 shows the general characteristics of the study population at baseline stratified for prevalent and incident depressive symptoms. Participants had a mean age of 59.6 ± 8.5 years on average, and 49.0% were women. Participants with prevalent or incident depressive symptoms had a lower education level, had a worse cardiovascular risk profile, and a less healthy lifestyle. Participants with prevalent depressive symptoms were significantly younger and were more often women. General characteristics of the study population stratified for persistent (*n* = 158), remitted (*n* = 61), and no depressive symptoms (*n* = 4045) are shown in online Supplementary Table S1.

**Table 1.** General characteristics of the study population according depression status

Characteristic	No depressive symptoms at baseline and follow-up (*n* = 4044)	Prevalent depressive symptoms (PHQ9 ⩾ 10) (*n* = 241)	Incident depressive symptoms (PHQ9 ⩾ 10) (*n* = 373)
Demographics			
Age (years)	59.6 ± 8.5	56.7 ± 8.7***	59.0 ± 8.8
Sex, *n* (% female)	1969 (48.7)	137 (56.8)*	196 (52.5)
Educational level, low/medium/high, *n* (%)	1208/1142/1694 (29.9/28.2/41.9)	105/72/64 (43.6/29.9/26.6)***	135/11/127 (36.2/29.8/34.0)**
Depression			
Depressive symptoms (PHQ-9 score)	1 [0–3]	12 [11–15]***	4 [2–7]***
Major depressive disorder (MINI), *n* (%)^a^	45 (1.1)	65 (28.3)***	27 (7.4)***
Anti-depressive medication, *n* (%)	192 (4.7)	64 (26.6)***	57 (15.3)***
Cardiovascular risk factors			
Waist circumference (cm)	93.3 ± 12.5	97.4 ± 14.8***	97.0 ± 14.4***
Office systolic BP (mmHg)	132.8 ± 17.3	133.2 ± 17.6	133.4 ± 17.7
Office diastolic BP (mmHg)	75.3 ± 9.5	76.3 ± 10.5	76.1 ± 9.7
Antihypertensive medication, *n* (%)	1285 (31.8)	108 (44.8)***	138 (37.0)*
Hypertension, *n* (%)	1970 (48.8)	135 (56.0)*	208 (55.8)*
Total-to-HDL cholesterol ratio	3.6 ± 1.1	3.8 ± 1.5**	3.7 ± 1.2
Lipid-modifying medication, *n* (%)	1079 (26.7)	78 (32.4)	118 (31.6)*
History of CVD, *n* (%)	474 (11.8)	39 (16.5)*	53 (14.4)
History of CVE^b^, *n* (%)	149 (3.7)	16 (6.8)*	15 (4.1)
T2DM, *n* (%)	739 (18.3)	74 (30.7)***	106 (28.4)***
Markers of CSVD			
Presence of lacunar infarct, *n* (%)	167 (4.1)	22 (9.1)**	11 (3.0)
Presence of cerebral microbleeds, *n* (%)	385 (9.7)	24 (10.3)	40 (11.0)
White matter hyperintensity volume (ml)	0.21 [0.07–0.70]	0.22 [0.06–0.61]	0.20 [0.08–0.63]
Intracranial volume (ml)	1395.2 ± 133.0	1353.3 ± 137.8**	1372.7 ± 135.0***
Life style factors			
Smoking, never/former/current, *n* (%)	1610/2014/417 (39.8/49.8/10.3)	84/103/52 (35.1/43.1/21.8)**	133/183/56 (35.8/49.2/15.1)*
Alcohol use, none/low/high, *n* (%)	606/2406/1028 (15.0/59.6/25.4)	70/128/41 (29.3/53.6/17.2)***	83/194/94 (22.4/52.3/25.3)*
Physical activity (hours/week)	14.2 ± 7.9	13.2 ± 8.7	13.3 ± 8.2*
Healthy diet (Dutch Healthy Diet Index)	84.8 ± 14.8	81.6 ± 15.1***	81.8 ± 16.0**
Markers of structural connectivity			
Global node degree	17.8 ± 0.4	17.7 ± 0.4	17.7 ± 0.3
Clustering coefficient	2.31 ± 0.08	2.31 ± 0.08	2.31 ± 0.08
Local efficiency	1.43 ± 0.14	1.44 ± 0.16	1.43 ± 0.15
Characteristic path length	0.84 ± 0.03	0.83 ± 0.03	0.83 ± 0.03
Global efficiency	1.49 ± 0.04	1.50 ± 0.04	1.50 ± 0.04

PHQ-9, indicates 9 item Patient Health Questionnaire; MINI, Mini-International Neuropsychiatric Interview; HbA1c, glycated hemoglobin A1c; HOMA-IR, Homeostasis Model Assessment of insulin resistance; BP, blood pressure; HDL, high-density lipoprotein; eGFR, estimated glomerular filtration rate; CVD, cardiovascular disease; CVE, cerebrovascular event; CSVD, cerebral small vessel disease; WMH, white matter hyperintensities; MRI, magnetic resonance imaging.

Prevalent and incident depressive symptoms are compared to no depressive symptoms. Data are presented as means ± standard deviation (s.d.), number (%) or median [interquartile range], and evaluated using independent *t* tests, Mann–Whitney *U* tests or χ^2^ tests (no depression *v.* prevalent depression and no depression *v.* incident depression).

a There can be a time lag between the assessment of the PHQ-9 and MINI.

b Self-report and MRI combined.

*p value < 0.05, **p value < 0.01, ***p value < 0.001.

Participants not included in the analyses (*n* = 2823, 33.1% of the total population due to missing MRI data), were on average older, lower educated, more depressed, had a worse cardiovascular risk profile, and a less healthy lifestyle than participants included in the analyses (*n* = 4866; data not shown).

### Associations of white matter connectivity with prevalent depression

Table 2 shows the associations of white matter dysconnectivity measures with prevalent depressive symptoms and MDD. More dysconnectivity, as reflected by a lower global node degree, was associated with higher prevalent depressive symptoms scores (PHQ-9 score) after adjustment for sex, age, education level, MRI date and T2DM in model 1 [rate ratio (RR; 95% confidence interval) 1.04 (1.00–1.07) per s.d.]. Although the RR remained almost similar [RR = 1.03 [1.00–1.07) per s.d.], this association became non-significant after further adjustment for cardiovascular risk factors in model 2. Furthermore, a lower global node degree was associated with prevalent clinically relevant depressive symptoms [PHQ-9 ⩾ 10; odds ratio (OR; 95% confidence interval) 1.21 (1.05–1.39) per s.d.], after full adjustment for demographic, cardiovascular, and lifestyle factors in model 3. While directionally similar, no significant association was found for global node degree with MDD [OR 1.12 (0.94–1.32) per s.d.]. In addition, no associations were found for the graph measures of white matter organization with prevalent depressive symptoms (PHQ-9 score or PHQ-9 ⩾ 10) or MDD (Table 2).

**Table 2.** Cross-sectional associations of white matter connectivity measures with prevalent depression

Model	Depressive symptoms (PHQ-9 score) Rate ratio (95% CI)	*p* value	Clinically relevant depressive symptoms (PHQ-9 ⩾ 10) *n* = 241 cases Odds ratio (95% CI)	*p* value	Major depressive disorder^a^ *n* = 143 cases Odds ratio (95% CI)	*p* value
*Number of connections*^b^						
Lower global node degree (per s.d.)						
Model 1	**1.04 (1.00–1.07)**	**0.041**	**1.19 (1.04–1.35)**	**0.010**	1.12 (0.94–1.32)	0.198
Model 2	1.03 (1.00–1.07)	0.087	**1.21 (1.06–1.38)**	**0.006**	1.14 (0.96–1.35)	0.145
Model 3	1.03 (0.99–1.07)	0.105	**1.21 (1.05–1.39)**	**0.007**	1.14 (0.96–1.35)	0.147
*White matter organization*^b^						
Lower clustering coefficient (per 1 s.d.)						
Model 1	1.00 (0.95–1.05)	0.880	1.03 (0.85–1.25)	0.757	1.18 (0.92–1.50)	0.197
Model 2	0.98 (0.93–1.03)	0.383	1.01 (0.83–1.23)	0.930	1.15 (0.89–1.47)	0.284
Model 3	0.98 (0.93–1.03)	0.414	1.01 (0.83–1.23)	0.941	1.16(0.90–1.48)	0.253
Lower local efficiency (per 1 s.d.)						
Model 1	1.00 (0.95–1.06)	0.966	1.02 (0.82–1.27)	0.854	1.27 (0.95–1.69)	0.103
Model 2	0.98 (0.92–1.04)	0.480	1.00 (0.79–1.25)	0.964	1.23 (0.92–1.64)	0.165
Model 3	0.98 (0.92–1.04)	0.497	0.99 (0.79–1.24)	0.945	1.24 (0.93–1.66)	0.147
Higher characteristic path length (per 1 s.d.)						
Model 1	1.00 (0.97–1.03)	0.875	1.06 (0.93–1.20)	0.382	0.99 (0.84–1.17)	0.931
Model 2	1.00 (0.96–1.03)	0.819	1.07 (0.94–1.22)	0.322	0.99 (0.84–1.18)	0.919
Model 3	1.00 (0.97–1.03)	0.907	1.07 (0.94–1.22)	0.331	0.99 (0.84–1.18)	0.940
Lower global efficiency (per 1 s.d.)						
Model 1	0.98 (0.95–1.02)	0.301	0.96 (0.84–1.10)	0.580	1.08 (0.91–1.28)	0.377
Model 2	0.98 (0.95–1.02)	0.294	0.97 (0.84–1.11)	0.635	1.10 (0.93–1.31)	0.263
Model 3	0.98 (0.95–1.02)	0.276	0.96 (0.84–1.10)	0.534	1.09 (0.92–1.30)	0.309

PHQ-9 indicates 9 item Patient Health Questionnaire; MINI, Mini-International Neuropsychiatric Interview; CI, confidence interval; SD, standard deviation.

PHQ-9 data *n* = 4866, MINI data *n* = 4707 in model 1. Cross-sectional data are evaluated using negative binominal regressions (PHQ-9 score) and logistic regressions (PHQ-9 ⩾ 10 and major depressive disorder).

**Model 1:** adjusted for global node degree (graph measures), age, sex, MRI date, educational level, and T2DM.

**Model 2:** additionally adjusted for waist circumference, total/high-density cholesterol ratio, lipid-modifying medication, systolic blood pressure, antihypertensive medication, history of cardiovascular disease, and history of cardiovascular accident. Data missing *n* = 63.

**Model 3:** additionally adjusted for smoking behavior and alcohol use. Additional data missing *n* = 3.

a Additionally adjusted for time between MINI assessment and MRI date.

b Global node degree, clustering coefficient, local efficiency, and global efficiency are inversed (i.e. multiplying it by − 1) to reflect structural dysconnectivity.

### Associations of white matter connectivity with incident depressive symptoms

Table 3 shows the associations of white matter connectivity measures with incident depressive symptoms. No association was found between global node degree and incident depressive symptoms compared to no depressive symptoms [model 1: HR = 1.05 (0.95–1.17) per s.d.]. Furthermore, no association was found for any of the graph measures with incident depressive symptoms. Kaplan-Meier curves showing the survival function for incident depressive symptoms by white matter connectivity measures are shown in online Supplementary Fig. S1.

**Table 3.** Longitudinal associations of white matter connectivity measures with incident depressive symptoms

Model	Incident depressive symptoms *n* = 373 cases Hazard ratio (95% CI)	*p* value
*Number of connections*^a^		
Lower global node degree (per s.d.)		
Model 1	1.05 (0.95–1.17)	0.344
Model 2	1.06 (0.95–1.19)	0.264
Model 3	1.06 (0.95–1.18)	0.334
*White matter organization*^a^		
Lower clustering coefficient (per 1 s.d.)		
Model 1	0.92 (0.79–1.07)	0.268
Model 2	0.91 (0.78–1.06)	0.202
Model 3	0.91 (0.78–1.06)	0.217
Lower local efficiency (per 1 s.d.)		
Model 1	0.93 (0.78–1.11)	0.403
Model 2	0.91 (0.76–1.08)	0.273
Model 3	0.91 (0.76–1.08)	0.282
Higher characteristic path length (per 1 s.d.)		
Model 1	0.99 (0.89–1.10)	0.806
Model 2	0.97 (0.87–1.08)	0.578
Model 3	0.97 (0.87–1.08)	0.550
Lower global efficiency (per 1 SD)		
Model 1	1.01 (0.91–1.12)	0.892
Model 2	1.00 (0.90–1.11)	0.985
Model 3	1.00 (0.90–1.11)	0.944

PHQ-9, indicates 9-item patient health questionnaire; CI indicates confidence interval; s.d., standard deviation. Incident depressive symptoms is defined as no depressive symptoms (PHQ-9 < 10) at baseline and depressive symptoms (PHQ-9 ⩾ 10) on at least one follow-up moment.

*n* = 4417 in model 1. Longitudinal data are evaluated using Cox proportional hazard regressions.

**Model 1:** adjusted for global node degree (graph measures), age, sex, MRI date, educational level, and T2DM.

**Model 2:** additionally adjusted for waist circumference, total/high-density cholesterol ratio, lipid-modifying medication, systolic blood pressure, antihypertensive medication, history of cardiovascular disease, and history of the cardiovascular accident. Data missing *n* = 50.

**Model 3:** additionally adjusted for smoking behavior and alcohol use. Additional data missing *n* = 2.

a Global node degree, clustering coefficient, local efficiency, and global efficiency are inversed (i.e. multiplying it by − 1) to reflect structural dysconnectivity.

### Associations of white matter connectivity with the course of depressive symptoms

The associations of white matter dysconnectivity measures with the course of depressive symptoms are shown in Table 4. A lower global node degree was associated with higher odds of a persistent course of depressive symptoms compared to no depressive symptoms [model 3: OR 1.21 (1.02–1.44) per s.d.). No association was found between global node degree and a remitted course of depressive symptoms compared to no depressive symptoms [model 1: OR 1.08 (0.83–1.41) per s.d.]. Furthermore, no associations were found between the graph measures and the course of depressive symptoms.

**Table 4.** Longitudinal associations of white matter connectivity measures with course of depressive symptoms

Model	Remitted depressive symptoms *n* = 61 cases Odds ratio (95% CI)	*p* value	Persistent depressive symptoms *n* = 158 cases Odds ratio (95% CI)	*p* value
*Number of connections*^a^				
Lower global node degree (per s.d.)				
Model 1	1.08 (0.83–1.41)	0.566	**1.21 1.03–1.42)**	**0.021**
Model 2	1.10 (0.83–1.45)	0.526	**1.22 (1.03–1.45)**	**0.019**
Model 3	1.11 (0.83–1.46)	0.489	**1.21 (1.02–1.44)**	**0.026**
*White matter organization*^a^				
Lower clustering coefficient (per 1 s.d.)				
Model 1	1.24 (0.84–1.82)	0.277	0.92 (0.72–1.16)	0.469
Model 2	1.32 (0.88–1.98)	0.180	0.85 (0.66–1.08)	0.184
Model 3	1.31 (0.87–1.97)	0.191	0.84 (0.66–1.08)	0.173
Lower local efficiency (per 1 s.d.)				
Model 1	1.36 (0.87–2.13)	0.181	0.89 (0.68–1.18)	0.426
Model 2	1.47 (0.91–2.36)	0.116	0.81 (0.61–1.08)	0.156
Model 3	1.46 (0.91–2.36)	0.119	0.81 (0.61–1.07)	0.142
Higher characteristic path length (per 1 s.d.)				
Model 1	1.08 (0.84–1.37)	0.556	1.04 (0.88–1.22)	0.660
Model 2	1.10 (0.86–1.41)	0.462	1.04 (0.88–1.22)	0.676
Model 3	1.10 (0.86–1.40)	0.463	1.04 (0.88–1.22)	0.663
Lower global efficiency (per 1 s.d.)				
Model 1	0.94 (0.73–1.22)	0.664	0.96 (0.82–1.13)	0.633
Model 2	0.93 (0.71–1.22)	0.618	0.96 (0.81–1.13)	0.612
Model 3	0.93 (0.71–1.22)	0.590	0.95 (0.80–1.12)	0.517

PHQ-9, indicates 9-item patient health questionnaire; CI indicates confidence interval; s.d., standard deviation. No depressive symptoms are defined as no depression at baseline and follow-up (PHQ-9 < 10), persistent depression as depression at baseline and follow-up (PHQ-9 ⩾ 10), and remitted depressive symptoms as depression at baseline (PHQ-9 ⩾ 10) and no depression during follow-up (PHQ-9 < 10).

Reference category no depressive symptoms (*n* = 4045). Longitudinal data are evaluated using multinomial logistic regressions.

**Model 1:** adjusted for global node degree (graph measures), age, sex, MRI date, educational level, and T2DM.

**Model 2:** additionally adjusted for waist circumference, total/high-density cholesterol ratio, lipid-modifying medication, systolic blood pressure, antihypertensive medication, history of cardiovascular disease, and history of cardiovascular accident. Data missing *n* = 53.

**Model 3:** additionally adjusted for smoking behavior and alcohol use. Additional data missing *n* = 3.

a Global node degree, clustering coefficient, local efficiency, and global efficiency are inversed (i.e. multiplying it by − 1) to reflect structural dysconnectivity.

### Additional analyses

Additional analyses are shown in online Supplementary Tables S2–S6. Adjustments to reduce potential misclassification of participants with subthreshold depression did not materially change the association of global average node degree with prevalent clinically relevant depressive symptoms (PHQ-9 ⩾ 10) and a persistent course of depressive symptoms. Furthermore, similar strengths of these associations were found after additional adjustment for healthy diet score, physical activity, white matter hyperintensity volume or time between PHQ-9 and MRI assessment. No associations of regional node degrees with the prevalent, incident, and persistent depression were found, with exception of a higher node degree with incident depressive symptoms for the medial temporal lobe (online Supplementary Table S5).

## Discussion

This population-based study found an association between a lower global node degree, i.e., the brain-averaged number of neuronal connections, and prevalent clinically relevant depressive symptoms in mid- to late life. In addition, a lower global node degree was associated with a persistent course of depressive symptoms. These associations were independent of demographical, cardiovascular, and lifestyle-related factors. No association was found between a lower global node degree and incident depressive symptoms. Furthermore, no associations of global graph measures of white matter connectivity organization with depression were found. These results might suggest that global loss of white matter connections, rather than global efficiency of the white matter network contribute to the prevalence and persistence of depression but not to the incidence of depression.

To our knowledge, this is the first study that investigates the associations of the number and organization of white matter connections with prevalence, incidence and course of depression in the general population. This study provides evidence that the global number of white matter connections may be altered in the presence of depression. In contrast to our expectations, we did not find an association between the global number of white matter connections and incident depressive symptoms. Although we did find an association between a higher number of white matter connection in the medial temporal lobe with incident depressive symptoms, no other regional associations were found. It might be that a higher number of white matter connections in the medial temporal lobe contribute to the development of depression. Alternatively, this is a false finding. It may be that alterations of white matter connections do not cause depression, or there may be reverse causation, in which depression causes alterations in the white matter. Alternatively, the absence of an association between alterations in global white matter connections and incident depression might also be the result of attrition bias. The finding of an association with a persistent course of depressive symptoms may indicate that alterations of white matter connections may predispose to chronic depression or that chronic depression alters brain connectivity. Replication of our findings in other population-based cohorts in both directions is warranted. Though, causality cannot be concluded.

In contrast to node degree, we found no associations between global graph measures of white matter organization and depression, which is in line with two previous clinical studies that did not find abnormalities in global graph measures in patients with MDD (Korgaonkar et al., 2014; Qin et al., 2014). However, two other studies did report on disrupted global graph measures in patients with remitted geriatric depression (Bai et al., 2012; Li et al., 2017). Importantly, these studies did not report the association with global node degree. The variation in graph measures within our relatively young and healthy study population was small, with standard deviations ranging from ~2% to 10%, which a priori limits the potential to find significant associations. In addition, heterogeneity in methodology, including the design (population-based *v.* clinic-based), study population (acutely depressed *v.* remitted patients; levels of depression severity), use of different measures of structural connectivity, and assessed brain regions (i.e. subnetwork instead of whole brain network), might explain the differences in results (Bai et al., 2012; Korgaonkar et al., 2014; Li et al., 2017; Qin et al., 2014). This heterogeneity was also reported in a recent systematic review of brain connectivity in individuals with MDD (Helm et al., 2018), which concluded that MDD-related structural changes concern gray matter densities of limbic (e.g. amygdala and hippocampus) and frontal regions and the connectome, especially cortico-limbic networks, but also temporal, thalamic, and cerebellum structures (Helm et al., 2018).

Several mechanisms may explain the association of abnormalities in the global node degree with prevalent and persistent depression. First, MRI features of cerebral small vessel disease may explain the observed associations. Disruptions in the white matter connectome may be related to vascular lesions, and vascular lesions in the white matter may lead to depression via disruption of frontal-limbic systems involved in mood regulation or their modulating pathways (Aizenstein et al., 2016; Alexopoulos, 2006; Krishnan, Hays, & Blazer, 1997). Indeed, the association with node degree became non-significant in model 3 after additional adjustment for total white matter hyperintensity volume (*p* = 0.065). Given that the regression coefficient changed only slightly, we feel that this finding needs further research before drawing firm conclusions. Second, the association between abnormalities in white matter connectivity and depression may exist because depression represents an early manifestation of dementia (Brodaty & Connors, 2020). This explanation is supported by the association of abnormalities in the white matter connectivity with both depression (Gong & He, 2015) and dementia (Tuladhar et al., 2016). We did not additionally adjust for cognitive function, as this might introduce collider bias (Schisterman, Cole, & Platt, 2009). Third, cardiometabolic factors can be the mechanisms underlying white matter connectivity and depression, as both are related to white matter integrity (Wassenaar, Yaffe, van der Werf, & Sexton, 2019) and depression (Marazziti, Rutigliano, Baroni, Landi, & Dell'Osso, 2014). However, adjustments for cardiovascular risk factors did not largely change our results, except for the association with PHQ-9 scores at baseline. Fourth, clinical symptoms of depression may lead to alterations in white matter connectivity. Processes as rumination may increase the activation of brain regions involved in mood regulation, and consequently affect the plasticity of brain regions involved in emotional processing, visual mental imagery, and attentional control (Zhang et al., 2020). Also, changes in diet, physical activity, lack of cognitive stimulation and social interactions during (recurrent) depressive episodes may influence brain-derived neurotropic factor levels, synapse formation, oxidative stress and inflammation (Mintzer et al., 2019; Mora, 2013). In our analyses, we corrected for most somatic and lifestyle factors. Alternatively, it can be that some individuals have a neurobiological predisposition for depression, in which a lower number of white matter connections makes them more vulnerable for depression.

Strengths of our study include its large sample size and population-based longitudinal design; the blinded semiautomatically processing of diffusion MRI scans; the use of the MINI diagnostic interview to assess the prevalence of MDD; the annual assessment of the PHQ-9 to assess both incidence and course of depressive symptoms over a seven-year period; and the assessment of both number and organization of global white matter connectivity markers.

This study also has limitations. First, selection and/or attrition bias could have occurred because participants with more severe depression and greater comorbidity were less likely to undergo MRI and follow-up assessments. This may have led to an underestimation of our results (Honningsvåg, Linde, Håberg, Stovner, & Hagen, 2012). Second, no data on MDD after the baseline examination was available (Janssen et al., 2016). Third, we performed multiple analyses in which we did not correct for multiple testing. Correction for multiple testing reduces the chance of type 1 error at the cost of increasing the risk for type 2 error. The graph measures are correlated and based on the same shared risk factors and disease mechanisms. Although these reasons withhold us to correct for multiple testing, the correction for multiple testing remains a point of discussion, and therefore, we underscore the importance of replication of our results in other future studies. Fourth, although the sample size was larger than previous studies, it is likely that the analyses were still under-powered (Marek et al., 2022).

Our findings contribute to a better understanding of the neuropathology underlying depression and might have important clinical implications. Plasticity of white matter connectivity has been demonstrated in several studies, including myelin formation and remodeling (Sampaio-Baptista & Johansen-Berg, 2017). Because of this reason, white matter connectivity may be a target for the prevention and treatment of (persistent) depression. Modifiable cardiovascular and lifestyle factors have been related to white matter connectivity. Cross-sectional studies have shown an association between white matter integrity and hypertension, obesity, T2DM, smoking, physical activity, cognitive training, diet, and meditation (Wassenaar et al., 2019). Preliminary evidence from cross-sectional studies of treated risk factors and interventional studies of protective factors, including physical activity, cognitive training, diet, and meditation, suggests that modification of these factors may impact white matter integrity (Wassenaar et al., 2019), which may consequently contribute to the prevention and treatment of depression.

In this population-based imaging study, fewer white matter connections were associated with prevalent depressive symptoms and a persistent course, but not incident depression. No associations between the global organization of white matter connections with prevalent, incident, or course of depression were found. Future studies are needed to replicate our findings.

## Supplementary material

For supplementary material accompanying this paper visit https://doi.org/10.1017/S0033291722002768. click here to view supplementary material
