# Supplementary material for: The association of white matter connectivity with prevalence, incidence and course of depressive symptoms: The Maastricht Study
Source: Psychol Med. 2022 Sep 7;53(12):5558–68. doi: 10.1017/S0033291722002768 (PMC10493191; doi:10.1017/S0033291722002768)
Supplement: Supplementary file 1 [file S0033291722002768sup001.docx]

**Supplemental Material**

**The Association Of White Matter Connectivity With Prevalence, Incidence, And Course Of Depressive Symptoms - The Maastricht Study**

Anouk FJ Geraets, PhD^1,2,3,6,7^, Sebastian Köhler, PhD^1,2,6^, Laura WM Vergoossen, PhD^1,4,6^, Walter H Backes, PhD^4,6^, Coen D.A. Stehouwer, MD, PhD^3,7^, Frans RJ Verhey, MD, PhD^1,2,6^, Jacobus FA Jansen, PhD^4,6^, Thomas T van Sloten, MD, PhD^3,7^ & Miranda T Schram, PhD^1,3,5,6,7^

^1^Department of Psychiatry and Neuropsychology, ^2^Alzheimer Centrum Limburg, ^3^Department of Internal Medicine, ^4^Department of Radiology and Nuclear Medicine, ^5^Heart and Vascular Center, Maastricht University Medical Center+ (MUMC+), Maastricht, the Netherlands; ^6^School for Mental Health and Neuroscience, ^7^School for Cardiovascular Diseases (CARIM), Faculty of Health, Medicine & Life Sciences, Maastricht University, Maastricht, the Netherlands

**Supplemental Methods**

***Brain Magnetic Resonance Imaging***

A 3D T1–weighted magnetization prepared rapid acquisition gradient echo (MPRAGE) sequence (TR/TI/TE 2,300/900/2.98 ms, 176 slices, 256×240 matrix size, and 1.00 mm cubic voxel size) was acquired for anatomic reference. dMRI data were acquired using a diffusion-sensitized echo-planar imaging (EPI) sequence (TR/TE 6,100/57 ms, 65 slices, 100×100 matrix size, 2.00 mm cubic voxel size, and 64 diffusion-sensitizing gradient directions [b=1,200 s/mm^2^]). In addition, three minimally diffusion-weighted images (b=0 s/mm^2^) were acquired.

***Image Pre-processing***

To define 94 regions, the automatic anatomical labelling 2 (AAL2) atlas (Tzourio-Mazoyer et al., 2002) was used. The AAL2 volumes of interest were transformed to diffusion space for each individual subject. First, affine registrations of the dMRI image to the T1 image and of the T1 image to T1 MNI-152 standard space (Fonov et al., 2011) were performed. These two transformations were combined, and the inverse transformation matrix was applied to the AAL2 template. T1-weighted and fluid-attenuated inversion recovery (FLAIR) images were segmented by use of an ISO13485:2012-certified, automated method (which included visual inspection) (Vrooman et al., 2007; De Boer et al., 2009) into white matter, grey matter, cerebrospinal fluid, and white matter lesions. Detailed methods were previously described (van Agtmaal et al., 2018). dMRI data analysis was performed with the diffusion MR Toolbox Explore DTI version 4.8.6 (Leemans, Jeurissen, Sijbers, & Jones, 2009). The main pre-processing steps were eddy current–induced geometric distortions and head motion correction, and estimation of the diffusion tensor. After preprocessing, fiber orientation distributions (FODs) were estimated using constrained spherical deconvolution with a maximum harmonic degree of 8 (Tournier, Calamante, & Connelly, 2013), which allows fiber tracking through regions with crossing fibers. Whole-brain deterministic tractography was performed using FOD sampling (Jeurissen, Leemans, Jones, Tournier, & Sijbers, 2011) with a seed point resolution of 2 mm3, a step size of 1 mm, and an FOD and maximum deflection angle threshold of 0.1 and 30°, respectively. The next step was performing connectivity analysis to obtain white matter tracts from and-to-all the segmented regions. A previous study of our group confirmed the robustness of tract volume as a measure for the edge weighting (Vaessen et al., 2012). Therefore, for each connection, the tract volume was calculated as the number of voxels visited by at least one tract between concerned areas multiplied by the voxel volume (in mm3). The obtained connectivity matrix with tract volumes was normalized to intracranial volume to reduce inter-subject variation (Whitwell, Crum, Watt, & Fox, 2001). When regions were connected by only one or two streamlines, the corresponding tract volumes were removed from the connectivity matrix, as an additional noise filter.

***Thresholding***

One structural group-averaged connectome was calculated from all individual structural connectomes (De Jong et al., 2019). For the structural group-averaged connectome, the individual connectomes were used in binarized form (relative tract volume > 0). To minimize the effect of spurious connections, the group-averaged connectomes was proportionally thresholded to a default sparsity of 0.80, meaning that only the connections that were present in at least 80% of the participants were taken into account in the individual structural connectivity analyses. 80% is a commonly used level of proportional thresholding from the literature (Buchanan et al., 2020). Before thresholding the individual connectomes with the group-averaged connectome (Vasa, Bullmore, & Patel, 2017), the participant’s structural overall connectivity was calculated as the mean from all weights in the structural connectivity (Van den Heuvel et al., 2017). Subsequently, each participant’s connectome was masked by the group-averaged connectome, resulting in a weighted, undirected network with a sparsity close to the sparsity of the group-averaged connectome.

***Depression***

Depressive symptoms were assessed by a validated Dutch version of the PHQ-9 (Kroenke, Spitzer, & Williams, 2001) both at baseline and during annual follow-up over seven years. The PHQ-9 is a self-administered questionnaire that assesses the presence of the nine symptoms for MDD on a 4-point Likert-scale ranging from 0 “not at all” to 4 ”nearly every day” (American Psychiatric Association, 1994). When one or two items were missing, the total score was calculated as 9×(total points/9−number of missing items) and rounded to the nearest integer. When more items were missing, the total score was scored as missing

**Supplemental Tables**

**Table S1. General characteristics of the study population according depression course**

| Characteristic | No depressive symptoms (n=4,045) | Remitted depressive symptoms (n=61) | Persistent depressive symptoms (n=158) |
| --- | --- | --- | --- |
| Demographics |  |  |  |
| Age (years) | 59.6±8.5 | 57.3±8.0* | 56.3±8.7*** |
| Sex, n (% female) | 1,969(48.7) | 31(50.8) | 98(62.0)** |
| Educational level,  low/medium/high, n (%) | 1,209/1,142/1,694  (29.9/28.2/41.9) | 22/22/17  (36.1/36.1/27.9) | 68/47/43  (43.0/29.7/27.2)*** |
| Depression |  |  |  |
| Depressive symptoms (PHQ-9 score) | 1.0[0.0-3.0] | 11.0[10.0-12.5]*** | 13.0[11.0-17.0]*** |
| Major depressive disorder (MINI), n (%) ^a^ | 45(1.1) | 10(16.9)*** | 48(31.8)*** |
| Anti-depressive medication, n (%) | 192(4.7) | 7(11.5)* | 52(32.9)*** |
| Cardiovascular risk factors |  |  |  |
| Body mass index (kg/m^2^) | 26.3±4.0 | 27.4±4.8* | 28.6±5.2*** |
| Waist circumference (cm) | 93.3±12.5 | 96.5±14.1 | 97.6±14.9*** |
| Office systolic BP (mmHg) | 132.8±17.3 | 132.3±16.9 | 132.9±17.8 |
| Office diastolic BP (mmHg) | 75.3±9.6 | 76.0±10.7 | 76.3±10.7 |
| Antihypertensive medication, n (%) | 1,285(31.8) | 23(37.7) | 77(48.7)*** |
| Hypertension, n (%) | 1,971(48.8) | 32(52.5) | 90(57.0) |
| Total-to-HDL cholesterol ratio | 3.6±1.1 | 3.7±1.1 | 3.8±1.5** |
| Triglycerides (mmol/l) | 1.3±0.8 | 1.4±0.9 | 1.6±1.2*** |
| Lipid-modifying medication, n (%) | 1,079(26.7) | 12(19.7) | 56(35.4)* |
| eGFR (ml/min/1.73m^2^) | 88.8±14.0 | 87.8±19.9 | 90.6±15.8 |
| Albuminuria,  normal/micro/macro, n (%) | 3,763/251/16  (93.4/6.2/0.4) | 56/4/1  (91.8/6.6/1.6) | 141/14/1  (90.5/8.9/0.6) |
| History of CVD, n (%) | 474(11.8) | 7(11.9) | 30(19.4)** |
| History of CVE ^a^, n (%) | 149(3.7) | 3(5.1) | 13(8.4)** |
| HbA1c (mmol/mol) | 38.4±8.3 | 39.3±8.7 | 42.5±13.5*** |
| Type 2 diabetes mellitus, n (%) | 739(18.3) | 16(26.2) | 53(33.5)*** |
| Markers of CSVD |  |  |  |
| Presence of lacunar infarct, n (%) | 167(4.1) | 5(8.2) | 13(8.2)* |
| Presence of cerebral microbleeds, n (%) | 385(9.7) | 5(8.5) | 17(11.0) |
| White matter hyperintensity volume (ml) | 0.21[0.07-0.70] | 0.27[0.10-0.50] | 0.20[0.05-0.62] |
| Intracranial volume (ml) | 1,395.2±133.0 | 1,380.1±146.0 | 1,339.9±136.0*** |
| Life style factors |  |  |  |
| Smoking,  never/former/current, n (%) | 1,610/2,014/418  (39.8/49.8/10.3) | 24/30/7  (39.3/49.2/11.5) | 56/61/39  (35.9/39.1/25.0)** |
| Alcohol use,  none/low/high, n (%) | 606/2,407/1,028  (15.0/59.6/25.4) | 10/44/6  (16.7/73.3/10.0)* | 52/74/31  (33.1/47.1/19.7)*** |
| Physical activity (hours/week) | 14.2±7.9 | 13.5±7.4 | 12.9±8.9 |
| Healthy diet (Dutch Healthy Diet Index) | 84.8±14.8 | 81.3±15.4 | 82.3±14.6 |
| Markers of structural connectivity |  |  |  |
| Global node degree | 17.8±0.4 | 17.8±0.3 | 17.7±0.4 |
| Clustering coefficient | 2.31±0.08 | 2.30±0.07 | 2.31±0.08 |
| Local efficiency | 1.49±0.04 | 1.49±0.03 | 1.50±0.04 |
| Characteristic path length | 1.43±0.14 | 1.45±0.16 | 1.44±0.16 |
| Global efficiency | 0.84±0.03 | 0.84±0.03 | 0.83±0.03 |
| MRI after update 16 Feb 2015, n (%) | 1,954(48.3) | 38(62.3)* | 78(49.4) |

Remitted and persistent are compared to no depressive symptoms. Data are presented as means ± standard deviation (SD), number (%) or median [interquartile range], and evaluated using independent T-tests, Mann–Whitney U tests or χ2 tests (no depression versus prevalent depression and no depression versus incident depression). PHQ-9 indicates 9 item Patient Health Questionnaire; MINI, Mini-International Neuropsychiatric Interview; HbA1c, glycated hemoglobin A1c; HOMA-IR, Homeostasis Model Assessment of insulin resistance; BP, blood pressure; HDL, high-density lipoprotein; eGFR, estimated glomerular filtration rate; CVD, cardiovascular disease; CVE, cerebrovascular event; CSVD, cerebral small vessel disease; WMH, white matter hyperintensities, MRI, magnetic resonance imaging. No depressive symptoms are defined as no depression at baseline and follow-up (PHQ-9 <10), persistent depression as depression at baseline and follow-up (PHQ-9 ≥10), and remitted depressive symptoms as depression at baseline (PHQ-9 ≥10) and no depression during follow-up (PHQ-9 <10). ^a^ There can be a time lag between the assessment of the PHQ-9 and MINI. ^b^ Self-report and MRI combined. **p*-value <0.05, ***p*-value <0.01, ****p*-value <0.001.

**Table S2**. **Cross-sectional association of global node degree with prevalent depressive symptoms**

| **Model** | **Clinically relevant depressive symptoms (PHQ-9≥10)**  **n=230 cases** Odds ratio (95% CI) | *P-*value |
| --- | --- | --- |
| **Lower global node degree (per SD)** |  |  |
| Model 3 | **1.21(1.05;1.39)** | **0.007** |
| Model 3 + antidepressant medication | **1.20(1.04;1.38)** | **0.010** |
| Model 3 excl. antidepressant users from the control group (excluded data n=261) | **1.21(1.05;1.38)** | **0.007** |
| Model 3 excl. participants with MDD or missing MDD data from the control group (excluded data n=226) | **1.21(1.06;1.39)** | **0.006** |
| Model 3 + healthy diet score (missing data n=230) | **1.24(1.07;1.43)** | **0.003** |
| Model 3 + physical activity (missing data n=394) | **1.21(1.04;1.41)** | **0.012** |
| Model 3 + WMH and intracranial volume | **1.18(1.03;1.36)** | **0.019** |
| Model 3 + time between PHQ-9 and MRI assessment | **1.21(1.05;1.39)** | **0.007** |

Cross-sectional data are evaluated using logistic regressions. Global node degree is inversed (i.e., multiplying it by −1) to reflect structural dysconnectivity. PHQ-9 indicates 9 item Patient Health Questionnaire; CI, confidence interval; SD, standard deviation, MDD, major depressive disorder; MRI, magnetic resonance imaging, WMH, white matter hyperintensity. PHQ-9 data n=4,800 in model 3.

**Model 3:** adjusted for age, sex, MRI date, educational level, type 2 diabetes mellitus, waist circumference, total/high density cholesterol ratio, lipid-modifying medication, systolic blood pressure, antihypertensive medication, history of cardiovascular disease, history of cardiovascular accident, smoking behavior and alcohol use.

**Table S3**. **Longitudinal association of global node degree with course of depressive symptoms**

|  | **Reference category no depressive symptoms (n=3,999)** | | |  |  | |  |
| --- | --- | --- | --- | --- | --- | --- | --- |
| **Model** | **Remitted depressive symptoms**  **n=58 cases** Odds ratio (95% CI) | *P-*value | **Persistent depressive symptoms**  **n=151 cases**  Odds ratio (95% CI) | | | *P-*value | |
| **Lower global node degree (per SD)** |  |  |  | | |  | |
| Model 3 | 1.11(0.83;1.47) | 0.489 | **1.21(1.02;1.44)** | | | **0.026** | |
| Model 3 + antidepressant medication | 1.11(0.84;1.48) | 0.465 | **1.23(1.03;1.47)** | | | **0.024** | |
| Model 3 excl. antidepressant users (excluded data n=243) | 1.15(0.86;1.54) | 0.346 | 1.19(0.97;1.45) | | | 0.093 | |
| Model 3 excl. participants with MDD or missing MDD data from the control group (excluded data n=174) | 1.11(0.83;1.47) | 0.483 | **1.22(1.03;1.45)** | | | **0.024** | |
| Model 3 + healthy diet score (missing data n=178) | 1.06(0.79;1.44) | 0.683 | **1.29(1.07;1.54)** | | | **0.006** | |
| Model 3 + physical activity (missing data n=330) | 1.08(0.79;1.47) | 0.638 | **1.25(1.04;1.50)** | | | **0.019** | |
| Model 3 + WMH and intracranial volume | 1.09(0.81;1.45) | 0.574 | 1.18(0.99;1.41) | | | 0.065 | |
| Model 3 + time between PHQ-9 and MRI assessment | 1.11(0.83;1.47) | 0.482 | **1.21(1.02;1.44)** | | | **0.029** | |

Node degree is inversed (i.e., multiplying it by −1) to reflect structural dysconnectivity. Longitudinal data are evaluated using multinomial logistic regressions. PHQ-9 indicates 9 item Patient Health Questionnaire; CI, confidence interval; SD, standard deviation, MRI; magnetic resonance imaging; WMH, white matter hyperintensity. No depressive symptoms are defined as no depression at baseline and follow-up (PHQ-9 <10), persistent depression as depression at baseline and follow-up (PHQ-9 ≥10), and remitted depressive symptoms as depression at baseline (PHQ-9 ≥10) and no depression during follow-up (PHQ-9 <10).

**Model 3:** adjusted for age, sex, MRI date, educational level, type 2 diabetes mellitus, waist circumference, total/high density cholesterol ratio, lipid-modifying medication, systolic blood pressure, antihypertensive medication, history of cardiovascular disease, history of cardiovascular accident, smoking behavior and alcohol use.

**Table S4**. **Cross-sectional associations of regional lower node degree with prevalent depression**

| **Brain area** | **Depressive symptoms**  **(PHQ-9 score)**  Rate ratio (95% CI) | *P-*value | **Clinically relevant depressive symptoms (PHQ-9≥10)**  **n=241 cases** Odds ratio (95% CI) | *P-*value | **Major depressive disorder ^a^**  **n=143 cases**  Odds ratio (95% CI) | *P-*value |
| --- | --- | --- | --- | --- | --- | --- |
| **Limbic system ^b,c^ (per 1 SD)** |  |  |  |  |  |  |
| Model 1 | 1.00(0.97;1.03) | 0.967 | 0.95(0.83;1.09) | 0.452 | 0.98(0.82;1.16) | 0.790 |
| Model 2 | 1.00(0.97;1.04) | 0.992 | 0.97(0.84;1.11) | 0.626 | 1.02(0.86;1.21) | 0.835 |
| Model 3 | 1.00(0.96;1.03) | 0.864 | 0.95(0.83;1.09) | 0.487 | 1.00(0.84;1.19) | 0.986 |
| **Anterior cingulate cortex ^c^ (per 1 SD)** |  |  |  |  |  |  |
| Model 1 | 1.01(0.98;1.05) | 0.496 | 1.01(0.88;1.16) | 0.845 | 0.96(0.81;1.15) | 0.675 |
| Model 2 | 1.02(0.99;1.06) | 0.281 | 1.05(0.91;1.21) | 0.489 | 1.02(0.86;1.22) | 0.795 |
| Model 3 | 1.02(0.98;1.05) | 0.401 | 1.03(0.90;1.19) | 0.645 | 1.01(0.85;1.21) | 0.887 |
| **Medial temporal lobe ^c^ (per 1 SD)** |  |  |  |  |  |  |
| Model 1 | 1.00(0.97;1.03) | 0.991 | 1.04(0.91;1.19) | 0.546 | 1.02(0.86;1.21) | 0.794 |
| Model 2 | 1.00(0.97;1.03) | 0.935 | 1.04(0.91;1.19) | 0.568 | 1.04(0.88;1.23) | 0.657 |
| Model 3 | 1.00(0.96;1.03) | 0.824 | 1.03(0.90;1.18) | 0.645 | 1.03(0.87;1.22) | 0.735 |
| **Prefrontal cortex ^c^ (per 1 SD)** |  |  |  |  |  |  |
| Model 1 | **1.04(1.00;1.07)** | **0.044** | 1.10(0.96;1.27) | 0.159 | 1.13(0.95;1.34) | 0.185 |
| Model 2 | **1.04(1.00;1.07)** | **0.036** | 1.13(0.98;1.30) | 0.084 | 1.15(0.96;1.37) | 0.121 |
| Model 3 | 1.03(1.00;1.07) | 0.059 | 1.13(0.98;1.30) | 0.104 | 1.14(0.96;1.37) | 0.142 |

PHQ-9 data n=4,866, MINI data n=4,707 in model 1. Cross-sectional data are evaluated using negative binominal regressions (PHQ-9 score) and logistic regressions (PHQ-9≥10 and major depressive disorder). PHQ-9 indicates 9 item Patient Health Questionnaire; MINI, Mini-International Neuropsychiatric Interview; CI, confidence interval; SD, standard deviation.

**Model 1:** adjusted for age, sex, MRI date, educational level, and type 2 diabetes mellitus.

**Model 2:** additionally adjusted for waist circumference, total/high density cholesterol ratio, lipid-modifying medication, systolic blood pressure, antihypertensive medication, history of cardiovascular disease, and history of cardiovascular accident. Data missing n=63.

**Model 3:** additionally adjusted for smoking behavior and alcohol use. Additional data missing n=3.

^a^Additionally adjusted for time between MINI assessment and scan date.

^b^Amygdala, hippocampus, caudate nucleus, putamen, thalamus, globus pallidus, cingulate gyrus.

^c^ Global node degrees are inversed (i.e., multiplying it by −1) to reflect structural dysconnectivity.

**Table S5**. **Longitudinal associations of regional lower node degree with incident depressive symptoms**

| **Brain area** | **Incident depressive symptoms**  **n=373 cases** Hazard ratio (95% CI) | *P-*value |
| --- | --- | --- |
| **Limbic system ^a,b^ (per 1 SD)** |  |  |
| Model 1 | 0.99(0.89;1.10) | 0.819 |
| Model 2 | 1.00(0.89;1.11) | 0.927 |
| Model 3 | 0.99(0.89;1.10) | 0.838 |
| **Anterior cingulate cortex ^b^ (per 1 SD)** |  |  |
| Model 1 | 0.97(0.88;1.08) | 0.575 |
| Model 2 | 0.99(0.89;1.09) | 0.784 |
| Model 3 | 0.98(0.88;1.09) | 0.676 |
| **Medial temporal lobe ^b^ (per 1 SD)** |  |  |
| Model 1 | **0.90(0.82;1.00)** | **0.039** |
| Model 2 | **0.90(0.82;1.00)** | **0.041** |
| Model 3 | **0.90(0.82;1.00)** | **0.039** |
| **Prefrontal cortex ^b^ (per 1 SD)** |  |  |
| Model 1 | 1.06(0.96;1.18) | 0.269 |
| Model 2 | 1.06(0.95;1.18) | 0.274 |
| Model 3 | 1.06(0.95;1.18) | 0.293 |

n=4,417 in model 1. Longitudinal data are evaluated using Cox proportional hazard regressions. PHQ-9 indicates 9-item patient health questionnaire; CI indicates confidence interval. Incident depressive symptoms is defined as no depressive symptoms (PHQ-9<10) at baseline and depressive symptoms (PHQ-9≥10) on at least one follow-up moment

**Model 1:** adjusted for age, sex, MRI date, educational level, and type 2 diabetes mellitus.

**Model 2:** additionally adjusted for waist circumference, total/high density cholesterol ratio, lipid-modifying medication, systolic blood pressure, antihypertensive medication, history of cardiovascular disease, and history of cardiovascular accident. Data missing n=50.

**Model 3:** additionally adjusted for smoking behavior and alcohol use. Additional data missing n=2.

^a^Amygdala, hippocampus, caudate nucleus, putamen, thalamus, globus pallidus, cingulate gyrus.

^b^ Global node degrees are inversed (i.e., multiplying it by −1) to reflect structural dysconnectivity.

**Table S6**. **Associations of regional lower node degree with course of depressive symptoms**

| **Brain area** | **Remitted depressive symptoms**  **n=61 cases** Odds ratio (95% CI) | *P-*value | **Persistent depressive symptoms**  **n=158 cases**  Odds ratio (95% CI) | *P-*value |
| --- | --- | --- | --- | --- |
| **Limbic system ^a,b^ (per 1 SD)** |  |  |  |  |
| Model 1 | 1.14(0.87;1.49) | 0.347 | 0.88(0.74;1.03) | 0.118 |
| Model 2 | 1.15(0.87;1.51) | 0.339 | 0.90(0.76;1.06) | 0.209 |
| Model 3 | 1.14(0.86;1.51) | 0.354 | 0.87(0.74;1.04) | 0.117 |
| **Anterior cingulate cortex ^b^ (per 1 SD)** |  |  |  |  |
| Model 1 | 1.28(0.96;1.70) | 0.100 | 0.95(0.81;1.12) | 0.554 |
| Model 2 | 1.28(0.95;1.72) | 0.108 | 1.01(0.85;1.20) | 0.893 |
| Model 3 | 1.27(0.94;1.72) | 0.114 | 0.99(0.84;1.18) | 0.950 |
| **Medial temporal lobe ^b^ (per 1 SD)** |  |  |  |  |
| Model 1 | 1.26(0.96;1.67) | 0.102 | 0.97(0.82;1.14) | 0.714 |
| Model 2 | 1.22(0.92;1.62) | 0.172 | 0.97(0.83;1.15) | 0.754 |
| Model 3 | 1.22(0.91;1.62) | 0.182 | 0.96(0.81;1.13) | 0.622 |
| **Prefrontal cortex ^b^ (per 1 SD)** |  |  |  |  |
| Model 1 | 1.14(0.88;1.49) | 0.316 | 1.06(0.90;1.26) | 0.485 |
| Model 2 | 1.20(0.92;1.58) | 0.182 | 1.08(0.91;1.28) | 0.402 |
| Model 3 | 1.21(0.92;1.59) | 0.171 | 1.06(0.89;1.27) | 0.484 |

Reference category = no depressive symptoms (n=4,045). Longitudinal data are evaluated using multinomial logistic regressions. PHQ-9 indicates 9-item patient health questionnaire; CI indicates confidence interval. No depressive symptoms are defined as no depression at baseline and follow-up (PHQ-9 <10), persistent depression as depression at baseline and follow-up (PHQ-9 ≥10), and remitted depressive symptoms as depression at baseline (PHQ-9 ≥10) and no depression during follow-up (PHQ-9 <10).

**Model 1:** adjusted for age, sex, MRI date, educational level, and type 2 diabetes mellitus.

**Model 2:** additionally adjusted for waist circumference, total/high density cholesterol ratio, lipid-modifying medication, systolic blood pressure, antihypertensive medication, history of cardiovascular disease, and history of cardiovascular accident. Data missing n=53.

**Model 3:** additionally adjusted for smoking behavior and alcohol use. Additional data missing n=3.

^a^Amygdala, hippocampus, caudate nucleus, putamen, thalamus, globus pallidus, cingulate gyrus.

^b^ Node degrees are inversed (i.e., multiplying it by −1) to reflect structural dysconnectivity.

**Supplemental Figures**

**Figure S1a. Kaplan-Meier curves for the association of global node degree with incident depression**


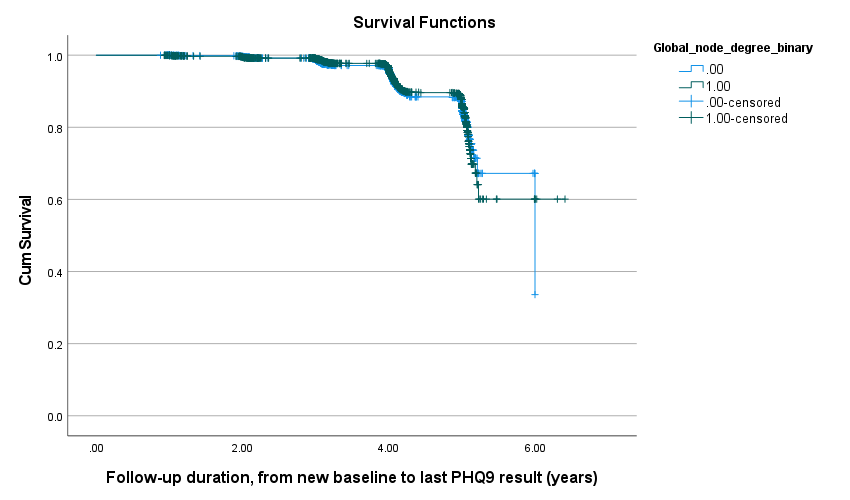


**Global node degree**

< mean

≥ mean

< mean-censored

≥ mean censored

**Figure S1b. Kaplan-Meier curves for the association of clustering coefficient with incident depression**


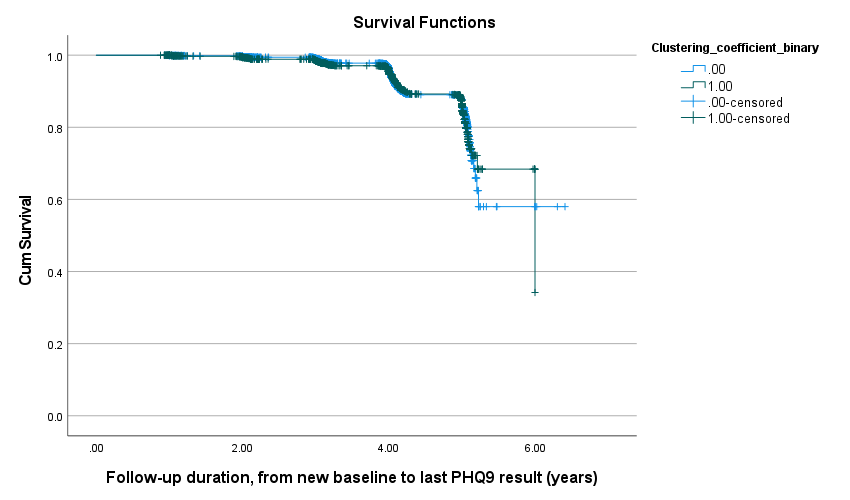


**Clustering coefficient**

< mean

≥ mean

< mean-censored

≥ mean censored

**Figure S1c. Kaplan-Meier curves for the association of local efficiency with incident depression**


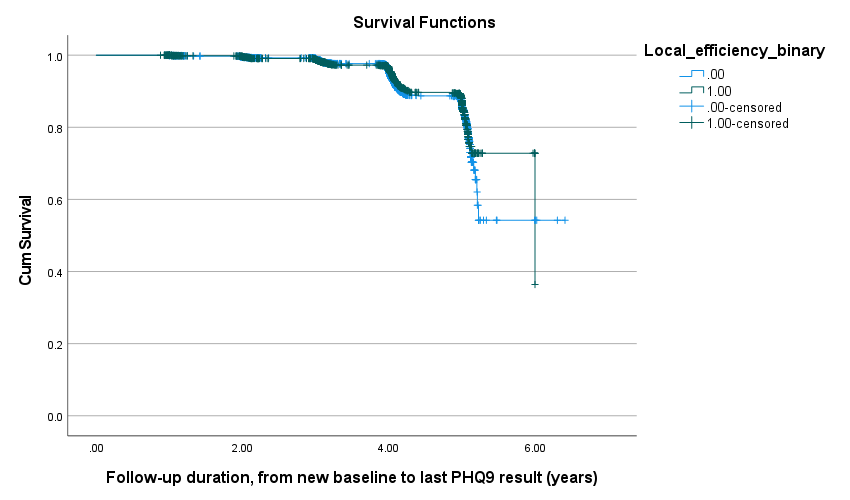


**Local efficiency**

< mean

≥ mean

< mean-censored

≥ mean censored

**Figure S1d. Kaplan-Meier curves for the association of characteristic pathlength with incident depression**


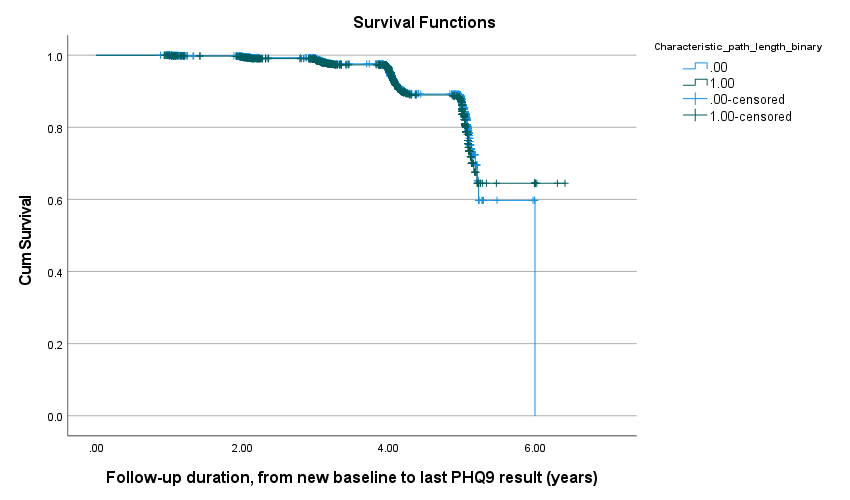


**Characteristic pathlength**

< mean

≥ mean

< mean-censored

≥ mean censored

**Figure S1e. Kaplan-Meier curves for the association of global efficiency with incident depression**


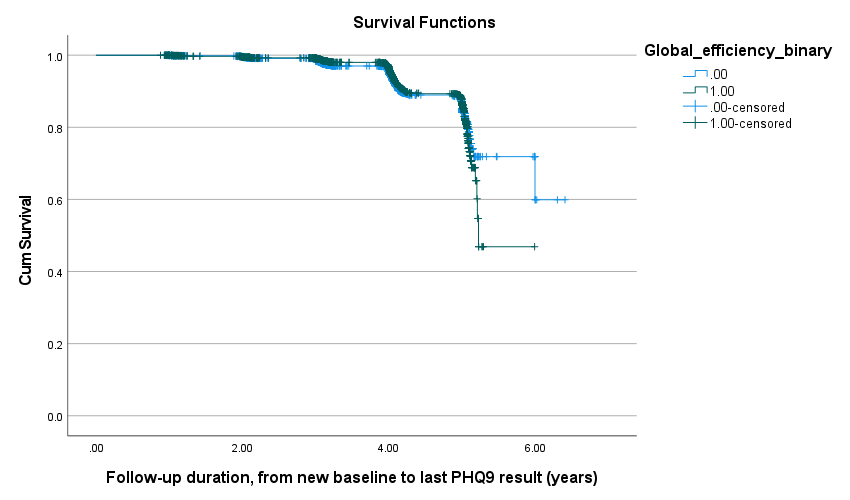


**Global efficiency**

< mean

≥ mean

< mean-censored

≥ mean censored

**Figure S2.** **Distribution of persistent and remitted depression over follow-up**


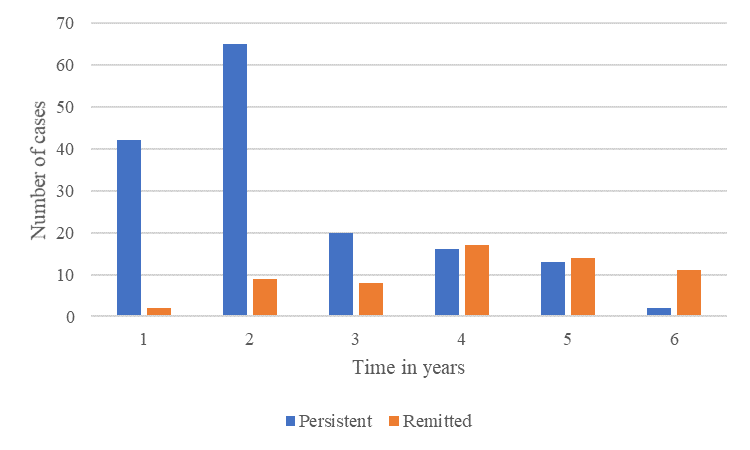


**References**

American Psychiatric Association. (1994). *Diagnostic and statistical manual of mental disorders* (4th ed.).

Washington, DC, American Psychiatric Association.

Buchanan, C. R., Bastin, M. E., Ritchie, S. J., Liewald, D. C., Madole, J. W., Tucker-Drob, … Cox, S. R.

(2020). The effect of network thresholding and weighting on structural brain networks in the

UK Biobank. *NeuroImage, 211,* 1-14. <https://doi.org/10.1016/j.neuroimage.2019.116443>.

De Boer, R., Vrooman, H. A., Van Der Lijn, F., Vernooij, M. W., Ikram, M. A., Van Der Lugt, A., … Niessen,

W. J. (2009). White matter lesion extension to automatic brain tissue segmentation on MRI.

*NeuroImage*, *45*(4), 1151-1161. <https://doi.org/10.1016/j.neuroimage.2009.01.011>.

De Jong, J. J. A., Jansen, J. F. A., Vergoossen, L. W. M., Schram, M. T., Stehouwer, C. D. A., Wildberger, J. E.,

… Backes, W. H. (2019). The Effect of MR Image Quality on Structural and Functional Brain Connectivity: The Maastricht Study. *bioRxiv*, 1-41. <https://doi.org/10.1101/806075>.

Fonov, V., Evans, A. C. , Botteron, K., Almli, C.R., McKinstry, R.C., & Collins, D.L. (2011). Unbiased

average age-appropriate atlases for pediatric studies. *NeuroImage*, *54*(1), 313-327.

<https://doi.org/10.1016/j.neuroimage.2010.07.033>.

Jeurissen, B., Leemans, A., Jones, D. K., Tournier, J. D., & Sijbers, J. (2011). Probabilistic fiber tracking using

the residual bootstrap with constrained spherical deconvolution. *Human Brain Mapping*, *32*(3), 461-

479. <https://doi.org/10.1002/hbm.21032>.

Kroenke, K., Spitzer, R., & Williams, J. (2001). The PHQ-9: Validity of a brief depression severity measure.

*The Journal of General Internal Medicine*, *16*(9), 606-613. <https://doi.org/10.1046/j.1525-1497.2001.016009606.x>.

Leemans, A., Jeurissen, B., Sijbers, J., & Jones, D. (2009). ExploreDTI: a graphical toolbox for processing,

analyzing, and visualizing diffusion MR data. *Proceedings of the International Society for Magnetic*

*Resonance in Medecine*, *17*, 3537. Retrieved from

<http://www.exploredti.com/ref/ExploreDTI_ISMRM_2009.pdf>

Tournier, J. D., Calamante, F., & Connelly, A. (2013). Determination of the appropriate b value and number of

gradient directions for high‐angular‐resolution diffusion‐weighted imaging. *NMR in Biomedicine*,

*26*(12), 1775-1786. <https://doi.org/10.1002/nbm.3017>.

Tzourio-Mazoyer, N., Landeau, B., Papathanassiou, D., Crivello, F., Etard, O., Delcroix N., … Joliot, M.

(2002). Automated anatomical labeling of activations in SPM using a macroscopic anatomical

parcellation of the MNI MRI single-subject brain. *NeuroImage*, *15*, 273-289.

<https://doi.org/10.1006/nimg.2001.0978>.

Vasa, F., Bullmore, E. T., & Patel, A.X. (2017). Probabilistic thresholding of functional connectomes:

Application to schizophrenia. *NeuroImage*, *172*, 326-340.

<https://doi.org/10.1016/j.neuroimage.2017.12.043>.

Vaessen, M. J., Jansen, J. F., Vlooswijk, M. C., Hofman, P. A., Majoie, H. M., Aldenkamp, A. P., … Backes,

W. H. (2012). White matter network abnormalities are associated with cognitive decline in chronic

epilepsy. *Cerebral Cortex*, *22*, 2139-2147. <https://doi.org/10.1093/cercor/bhr298>.

van Agtmaal, M. J., Houben, A. J., de Wit, V., Henry, R. M., Schaper, N. C., Dagnelie, P. C., … Stehouwer, C.

D. A. (2018). Prediabetes Is Associated With Structural Brain Abnormalities: The Maastricht Study.

*Diabetes Care*, *41,* 2535-2543. <https://doi.org/10.2337/dc18-1132>.

Van den Heuvel, M. P., de Lange, S. C., Zalesky, A., Seguin, C., Yeo, B. T. T., & Schmidt, R. (2017).

Proportional thresholding in resting-state fMRI functional connectivity networks and consequences for

patient-control connectome studies: Issues and recommendations. *NeuroImage,* *152,* 437-449. <https://doi.org/10.1016/j.neuroimage.2017.02.005>.

Vrooman, H. A., Cocosco, C. A., van der Lijn, F., Stokking, R., Ikram, M. A., Vernooij, M. W., ... Niessen, W.

J. (2007). Multi-spectral brain tissue segmentation using automatically trained k-Nearest-Neighbor

classification. *NeuroImage*, *37*(1)*,* 71-81. <https://doi.org/10.1016/j.neuroimage.2007.05.018>.

Whitwell, J. L., Crum, W. R., Watt, H. C., & Fox, N. C. (2001). Normalization of cerebral volumes by use of

intracranial volume: implications for longitudinal quantitative MR imaging. *American Journal of*

*Neuroradiology*, *22*(8), 1483-1489. Retrieved from <http://www.ajnr.org/content/22/8/1483.long>
